# Deep learning-based electrical impedance spectroscopy analysis for malignant and potentially malignant oral disorder detection

**DOI:** 10.1038/s41598-025-05116-8

**Published:** 2025-06-03

**Authors:** Zhicheng Lin, Zi-Qiang Lang, Lingzhong Guo, Dawn C Walker, Malwina Matella, Mengxiao Wang, Craig Murdoch

**Affiliations:** 1 School of Electrical and Electronic Engineering, University of Sheffield, Sheffield, UK; 2 Insigneo, University of Sheffield, Sheffield, UK; 3 School of Computer Science, University of Sheffield, Sheffield, UK; 4 School of Clinical Dentistry, University of Sheffield, Sheffield, UK; 5 School of Electrical and Electronic Engineering, Amy Johnson Building, Portobello Street, Sheffield, S1 3 JD UK

**Keywords:** Deep learning, Electrical impedance spectroscopy, Oral cancer, Potentially malignant lesions, Dysplasia, Biomedical engineering, Oral cancer detection, Diagnostic devices

## Abstract

Electrical impedance spectroscopy (EIS) is a powerful tool used to investigate the properties of materials and biological tissues. This study presents one of the first applications of EIS for the detection and classification of oral potentially malignant disorders (OPMDs) and oral cancer. We aimed to apply EIS in conjunction with deep learning to assist the clinical diagnosis of OPMD and oral cancer as a non-invasive diagnostic technology. Currently, the diagnosis of OPMD and oral cancer relies on clinical examination and histopathological analysis of invasive scalpel tissue biopsies, which is stressful for patients, time-consuming for clinicians and subject to histopathological interobserver variation in diagnosis, although recent advances in artificial intelligence may circumvent discrepancy. Here we developed a novel deep learning convolutional neural network (CNN)-based method to automatically differentiate normal, OPMD and malignant oral tissues using EIS measurements. EIS readings were initially taken from untreated or glacial acetic acid-treated porcine oral mucosa and analyzed via CNN to determine if this method could discriminate between normal and damaged oral epithelium. CNN models achieved area under the curve (AUC) values of 0.92 ± 0.03, with specificity 0.95 and sensitivity 0.84, showing good discrimination. EIS data from ventral tongue and floor-of-the-mouth were collected from 51 healthy humans and 11 patients with OPMD and oral cancer. When a binary classification (low or high risk of malignancy) was applied, the best CNN model achieved an AUC 0.91 ± 0.1, with accuracy 0.91 ± 0.05, specificity 0.97 and sensitivity 0.74. These results demonstrate the considerable potential of EIS in combination with CNN models as an adjunctive non-invasive diagnostic tool for OPMD and oral cancer.

**Supplementary Information:**

The online version contains supplementary material available at 10.1038/s41598-025-05116-8.

## Introduction

Head and neck cancer is the seventh most prevalent cancer worldwide. A large proportion of these cancers constitute malignancies affecting the lip and oral cavity and are often termed oral squamous cell carcinoma (OSCC) or oral cancer. In 2020, there were 377,713 new cases of OSCC diagnosed with 177,757 deaths recorded^1^ with incidence increasing^2^. OSCC significantly impacts the patient’s quality of life and has a poor prognosis, where the overall 5-year survival is just 54%. However, early detection improves the chances of survival to 90%^3^. Traditionally, diagnosis of oral potentially malignant disorders (OPMD) and OSCC has relied on clinical examination followed by an invasive scalpel tissue biopsy, which is subjected to histopathological examination by a specialist pathologist^4^. Histopathological analysis is subjective, with research showing much variability in the grading of OPMD between pathologists^5^. Although recent advances in OPMD and oral cancer diagnosis based on machine learning and convolutional neural networks (CNN) applied to histological images have shown improved consistency, aiding diagnosis and prognostic prediction^6,7^, these technologies still require the acquisition of a scalpel biopsy. Consequently, there is a need for non-invasive diagnostic tools that can aid in the early detection and monitoring of OPMD and oral cancer negating the need for repeat invasive biopsy.

Electrical impedance spectroscopy (EIS) is a powerful tool to investigate the properties of materials and biological samples, and has been used in a variety of applications, including food quality monitoring^8^, bacterial detection^9^, assessment of preterm birth^10^, bone healing after fracture^11^, skin layer assessment^12^, position measurement^13^ and for some cancers^14,15^. The electrical properties of a tissue are highly dependent on its physiological structure along with its molecular and cellular composition^16^. EIS spectra have been successfully used to distinguish between normal, potentially malignant and cancer in both cervical^17,18^ and oral mucosal^19^ tissue. In fact, use of EIS for the detection of cervical intraepithelial neoplasia as an adjunctive to colposcopy is now used in some clinical practices^20^, whereas progress toward clinical use for oral lesions has been slow. Several reasons may explain this, such as the increased structural complexity of the oral compared to cervical epithelium and the more varied molecular pathways to carcinogenesis giving rise to heterogeneous tissue structures, meaning that OPMD are often difficult to discriminate with EIS readings alone^19^. To address this, application of EIS-based deep learning models such as CNNs, may facilitate a more nuanced approach in the early detection of OPMD and therefore cancer risk, moving beyond the conventional use of EIS readings to offer a more quantitative characterization, allowing the enhanced discrimination of normal, OPMD and OSCC tissue, thereby improving diagnostic capabilities.

EIS is a non-invasive method that measures the impedance of biological tissues at multiple frequencies, providing valuable insights into the tissue composition and structural changes of healthy oral mucosa and their associated lesions as disease progresses. The impedance values obtained at different frequencies can be used as independent inputs for feature extraction through a CNN network. Through convolutional operations, the network can automatically learn the relationship between various EIS spectra to enhance the understanding of the features of the EIS data. Then, after training, the CNN can be used as a classifier to evaluate the risk of oral cancer.

A neural network consists of multiple layers of nodes, including an input layer, one or more hidden layers, and an output layer. Each node (neuron) is connected to another node with associated weights and thresholds. During the data training process, through back-propagation, the weights of each node are updated until certain conditions are met, such as achieving a sufficiently low value of the loss function or convergence to a specific value, indicating that the model training has been completed. In this study, one-dimensional convolutional layers were introduced before the neural network’s input layer to perform the feature extraction of the EIS spectra, with the hope of improving the model’s classification accuracy. Convolutional operations involve the summation of multiple convolution kernels applied to the original data, resulting in filtered new data that can enhance the relationships between adjacent data. Several studies have discussed the use of deep neural networks for analyzing EIS data. In Doonyapisut et al., deep learning networks were employed to extract meaningful information from simulated EIS spectra, where the EIS layer was composed of 100-D vectors with six channels for each spectrum^21^. Sun et al., proposed a deep learning-based method to analyse impedance spectra for the fully charged and fully discharged states of battery life using a constant-current curve^22^. The performance of CNN algorithms for the classification and detection of OPMD and OSCC using oral photographic images has previously been evaluated^23^. Compared with images, EIS spectra provide information from within tissue where tissue structures change as malignancy progresses. However, currently there is little research on using CNN and EIS data to aid in the diagnosis of OPMD and oral cancer.

In this study, we initially used normal and acetic acid-treated pig oral mucosa as a proof-of-concept experiment to show that EIS in conjunction with CNNs could differentiate between healthy and damaged oral epithelium. We next used the EIS-based CNN to differentiate between healthy oral mucosa, different grades (mild, moderate, severe) of OPMD and oral cancer. The model was trained and evaluated using 28 impedance (14 real parts and 14 imaginary parts) obtained at 14 frequencies (76 Hz to 625 kHz) from EIS data of the ventral tongue and floor-of-the-mouth of patients. The best CNN model achieved an AUC 0.907 ± 0.097, accuracy 0.905 ± 0.045, specificity 0.916 and sensitivity 0.743, demonstrating the potential use of EIS-based CNN models as an adjunctive non-invasive diagnostic tool for the early detection of OPMD and oral cancer.

## Materials and methods

### EIS data and measurement

Due to the presence of cellular, stromal, and other constituents, all biological tissues exhibit impedance characteristics that depend on frequency. The EIS at a particular frequency $\:\omega\:$ is a complex number represented by two components^8^: the real part $\:{Z}_{Re}$ and the imaginary part $\:{Z}_{Im}\:$as follows:

1 $$\:\begin{array}{c}Z\left(\omega\:\right)={Z}_{Re}\left(\omega\:\right)-j{Z}_{Im}\:\left(\omega\:\right)\end{array}$$

**Fig. 1:**
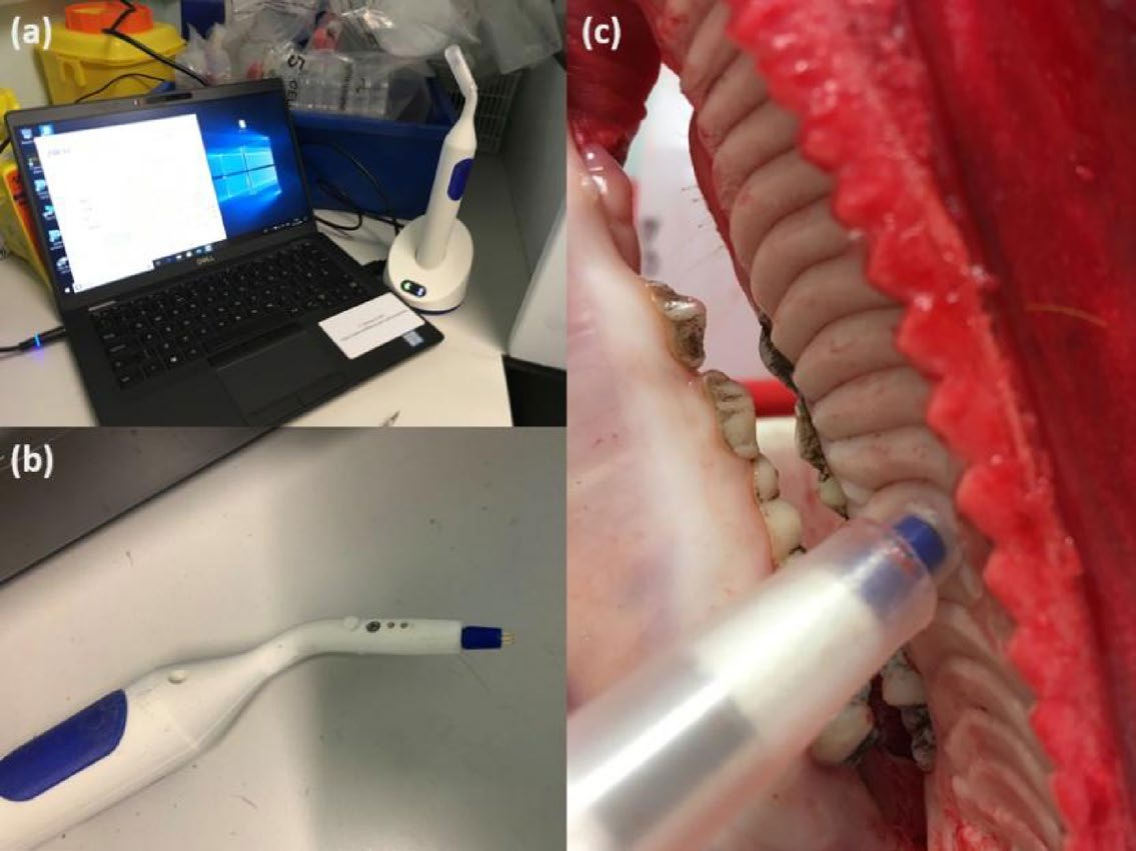
The electrical impedance spectroscopy measurement device and an illustration of how EIS reading was taken from porcine mucosal tissue. (**a**) EIS Hand-held device and base-station linked to a lap-top computer, which receives the EIS measurements via Bluetooth from the device following probing of tissue; (**b**) Hand-held device; (**c**) EIS probe in contact with the porcine hard palate in the mouth during the acquisition of an EIS reading from the tissue.

The EIS of oral tissue at different oral locations was measured using an EIS measurement device (Fig. 1a-b) over 14 frequencies ranging from 76 Hz to 625 kHz as previously described^19^. Each dataset was composed of 14 real and 14 imaginary parts. A commercial electrical impedance spectroscopy (EIS) system, ZedScan (Zilico Ltd., UK)^19^, was used for all experiments in this study. For oral measurements, a modified version of the ZedScan probe was utilized to better accommodate the anatomical structure of the oral cavity. This modified probe retained the same functional and electrical properties as the original design. The same device and impedance acquisition system were employed in both the porcine and human tissue experiments to ensure data comparability.

The EIS device consists of a handheld unit, a base-station that acts as a charging and data transfer point to a local computer where the data link to bespoke software. A disposable sheath covers the probe on the front end of the handheld unit (Fig. 1). During measurements, the tip with a sheath (diameter of 5.5 mm) was brought into contact with oral tissue. The probe’s tip contains four gold electrodes, uniformly spaced at a constant distance, allowing for specific current penetration into the tissue. Upon placing the electrodes on oral tissue and activating the hand-held device, a small electrical current was passed into the tissue. During this process, a current (12µA p-p) passes between adjacent pairs of electrodes and the voltage was measured between the remaining pair. The applied current covered a frequency range of 76 Hz to 625 kHz, with 14 binary steps, and the minimum time required to record the full spectrum was 20 ms. Data were captured in real-time and immediately downloaded to a computer via Bluetooth for analysis.

All measurements were carried out in accordance with relevant guidelines and regulations by the ethics committee of University of Sheffield. And all experimental protocols were approved by University of Sheffield. Informed consent was obtained from all subjects.

### Porcine data collection

Porcine mucosa closely resembles that of human oral mucosa^24^. Therefore, in the proof-of-concept experiments, EIS readings were taken from the ventral tongue (VT) and floor of the mouth (FM) of normal porcine mucosa (obtained from a local abattoir; *n* = 15 pig head, 220 EIS readings in total) and from the same tissue following treatment with glacial acetic acid for 5 min at room temperature (*n* = 3 pig heads, 80 EIS readings in total) to mimic damaged/diseased oral mucosa. Although no direct histological or molecular validation was performed in this study, acetic acid has been well-documented in previous studies^25,26^ to cause epithelial and mucosal injury in porcine models. This approach is commonly used to simulate intestinal damage in experimental settings. In our experiments, the application of acetic acid produced measurable changes in EIS signals, indirectly reflecting underlying tissue alterations and validating its use as a practical model for assessing the device’s performance.

### Human data collection

**Fig. 2:**
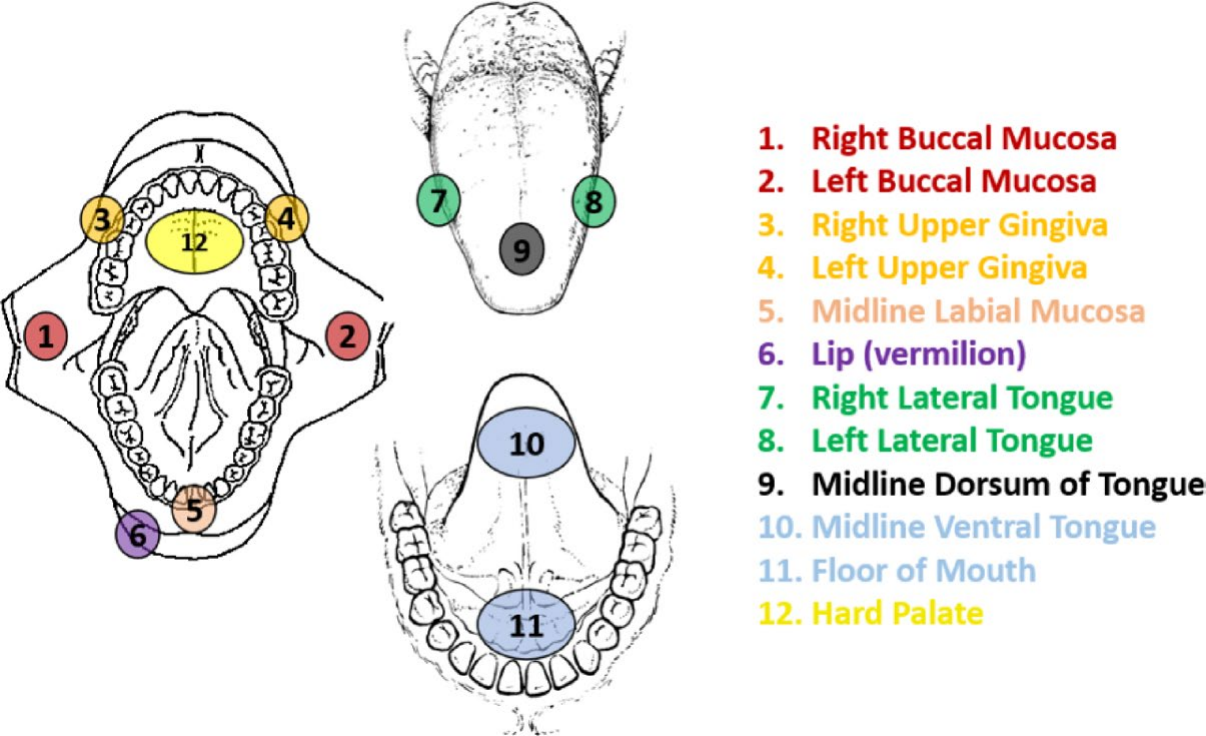
Schematic diagram showing the anatomical sites where electrical impedance spectroscopy measurements were sampled in subjects. Diagram adapted from^19^.

The human data consist of two parts: data from healthy volunteers with no previous diagnosis of oral mucosal disease and data from patients with oral lesions^19^. As control group, the EIS spectra of healthy volunteers (*n* = 51) were obtained from 12 different anatomical positions inside oral cavity (Fig. 2). The positions marked with the same colour correspond to oral sites that share similar histological properties in high-grade dysplasia, including epithelial thickness, keratinization pattern, and underlying connective tissue features. The patient data include 47 study subjects which were recruited in a non-consecutive manner from patients attending the Oral Medicine clinic at the Charles Clifford Dental Hospital, Sheffield Teaching Hospitals National Health Service Foundation Trust, Sheffield, United Kingdom. Under the WHO classification system^27^, the patient data were histopathologically graded by a pathologist and categorized as: having oral squamous cell carcinoma (OSCC) (*n* = 10), severe (*n* = 7), moderate (*n* = 10), mild (*n* = 10) oral epithelial dysplasia (OED), a histopathological feature that may be present in oral potentially malignant disorders (OPMDs), but which does not define them exclusively^28^, and ten cases classified as non-dysplastic lesions, based on histopathological evaluation and supported by longitudinal clinical follow-up in which no malignant transformation was observed. This categorization reflects retrospective outcome data rather than a predictive assumption. The data were also classified using a binary classification system^28^ where the data were split into two groups: low-risk lesion group (*n* = 24 for low-grade dysplasia and non-dysplastic lesions) and a high-risk lesion group (*n* = 23, that includes high-grade dysplasia and cancer)^19^.

**Fig. 3:**
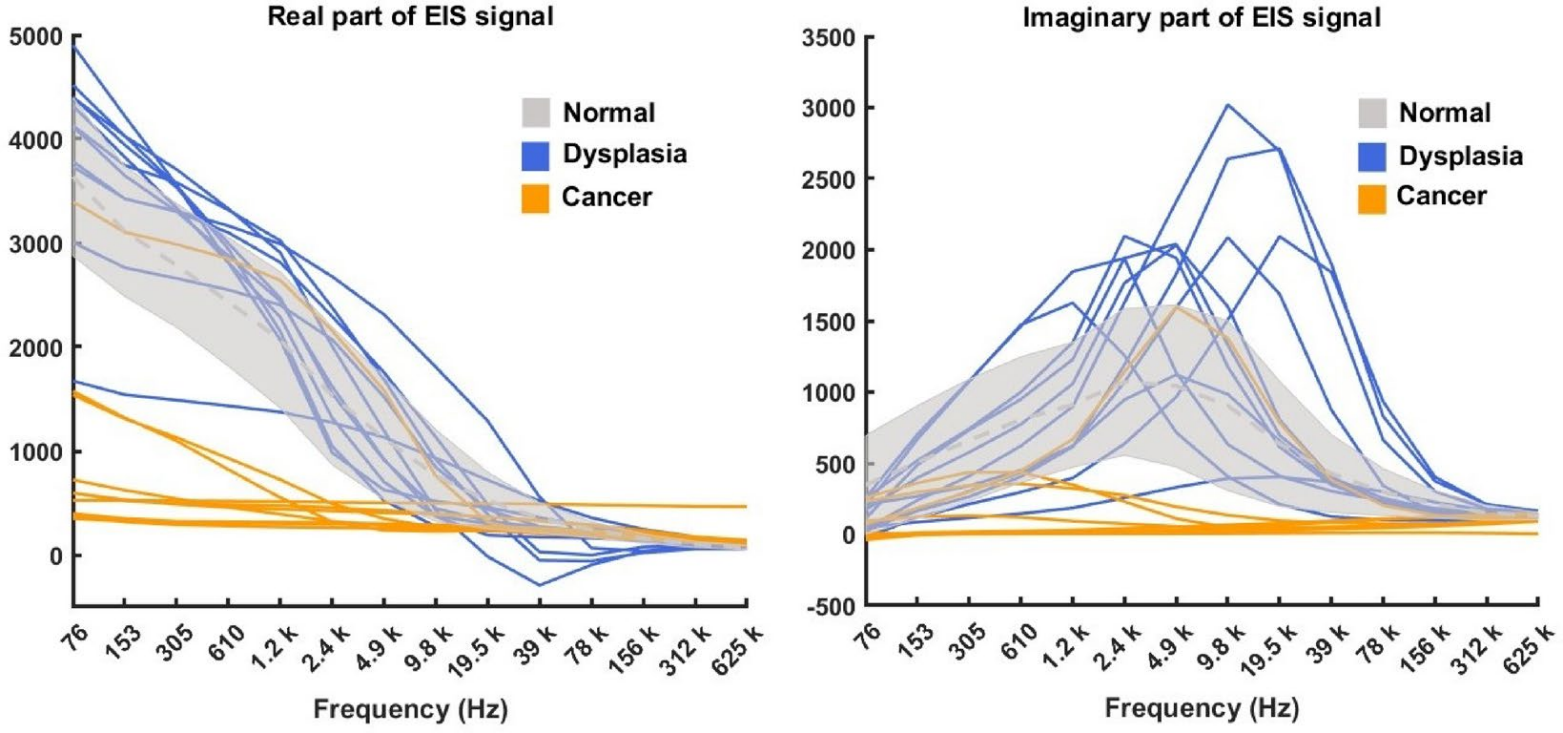
Visualization of the EIS spectra of the human data including normal (grey), histopathologically confirmed dysplasia (blue) and cancer (orange) cases. The grey area represents the distribution of normal healthy samples. The grey dashed line indicates the mean value, while the upper and lower boundary of the grey area are defined by the mean plus and minus the standard deviation. The blue and red curves represent dysplasia and cancer samples, respectively.

Since the epithelium of the VT and FM are very similar to each other in terms of epithelial keratinization (both are non-keratinised), thickness and structure, data for these two tissue types were combined to increase the sample size. Using the WHO classification system^27^, among 11 patients whose situation related to VT or FM, there were 1 case of mild dysplasia (with 2 readings), 3 cases of moderate dysplasia (with 4 readings), and 2 cases of severe dysplasia (with 5 readings), and 5 cases of cancer (with 10 readings). Using the binary classification method^28^, patients were split into 2 low-risk cases (with 3 readings) and 9 high-risk cases (with 18 readings). A summary of the data distribution used in this study under both the WHO^27^ and Binary classification standard^19,28^ is shown in Table 1 and visualized in Fig. 3. Some subjects had different numbers of EIS readings taken from their oral lesions; in this study, each reading was treated as an independent variable. Although healthy volunteer EIS spectra were collected from 12 anatomical sites, only data from the ventral tongue and floor of the mouth (i.e., non-keratinised tissues matching the lesion locations) were included in model training and evaluation. This approach ensured anatomical and histological consistency between healthy and lesion cases.

**Table 1:** Data statistical information.

Standard	Type	Number of subjects	Number of readings
WHO	Cancer	5	10
	Mild dysplasia	1	2
	Moderate dysplasia	3	4
	Severe dysplasia	2	5
	Healthy	51	102
Binary	Low-risk	2	3
	High-risk	9	18
	Healthy	51	102

**Fig. 4:**
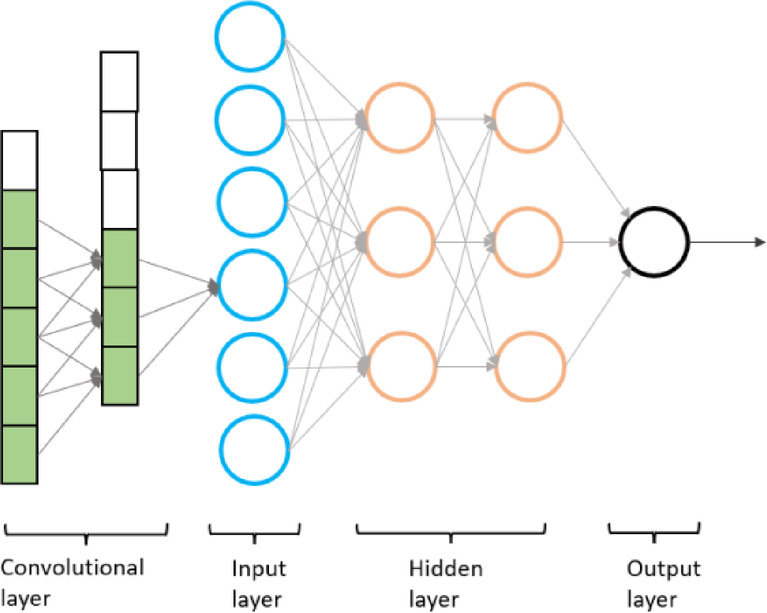
Structure of convolutional neural network (CNN).

### Convolutional neural networks

During the model selection, a basic neural network architecture was initially chosen, consisting of input, hidden, and output layers (Fig. 4). Given that each EIS spectrum comprised 28 data points (14 real and 14 imaginary), and considering the number of classes, the structures of the input and output layers were fixed. When setting the control group, variations were introduced by altering the number of neurons in the hidden layers and the number of layers in comparison. Furthermore, this study enhanced the basic CNN architecture by incorporating two additional one-dimensional convolutional layers for extracting high-dimensional feature information from the raw input data (Supplementary Table S1). This was done to investigate whether the convolutional operations could improve the model’s classification accuracy.

During the model training, considering the imbalance of the classes, e.g., between the cancer case (*n* = 18) and the healthy case (*n* = 100), the weighted Cross Entropy loss function as follows was used.

2 $$Obj = - \sum\limits_{c = 1}^M {{W_c}{y_c}log\left( {{p_c}} \right)} + \;\frac{\lambda }{2} * \sum\limits_{i = 1}^N {w_i^2}$$

where $\:{W}_{c}$ stands for the weight for the cth class, $\:{y}_{c}$ and $\:{p}_{c}$ represent the ground truth (true label) and the corresponding model predicted probability, respectively; $\:{w}_{i}$ represents the ith weight or bias in the model which was optimized during back-propagation; $\:\lambda\:$ denotes the weighting factor in L2 regularization, *M* denotes the number of the classes and *N* denotes the total number of weights and bias in the CNN.

In this loss function, by assigning a higher weight to a minority class, the model was encouraged to give more attention to it, to improve its ability to correctly classify instances from that class. To prevent overfitting, L2 regularization was also incorporated into the loss function to constrain model parameters. This was achieved by adding a penalty term with a weighting factor $\:\lambda\:$ to the loss function to discourage the model from assigning excessively large values to its parameters.

Overall, there were 5 hyper-parameters: number of hidden layers (2/3), number of neurons in hidden layers (125/250), with/without convolutional layers, the weight setting for each class in loss function and $\:\lambda\:$ (0.1/0.01) in L2 regularization. To facilitate clarity, the notation ‘Model_xl_yc_z’ is adopted to represent the structure of the applied CNN model where: ‘xl’ denotes the number of hidden layers, ‘yc’ denotes the number of one-dimensional convolutional layers with ‘c’ indicating convolution, ‘z’ denotes the number of neurons in each hidden layer. Additionally, the notation ‘W1:W2:W3 W’ is also used where Wi, i = 1,2,3, indicates the weights assigned to ith class in the weighted cross entropy loss function in Eq. (2) and W is the value of λ used for L2 regularization.

### Experimental design

The experiments consisted of 3 tasks. The first task used the pig-derived EIS data under the binary classification method (low/high-risk) to investigate the performance of CNNs in distinguishing normal and abnormal porcine tissues. The second and third tasks used the EIS data from healthy human and patients graded under the WHO and binary classification method (low/high-risk), respectively, to evaluate and investigate the feasibility of EIS based deep learning models for oral cancer diagnosis.

**Fig. 5:**
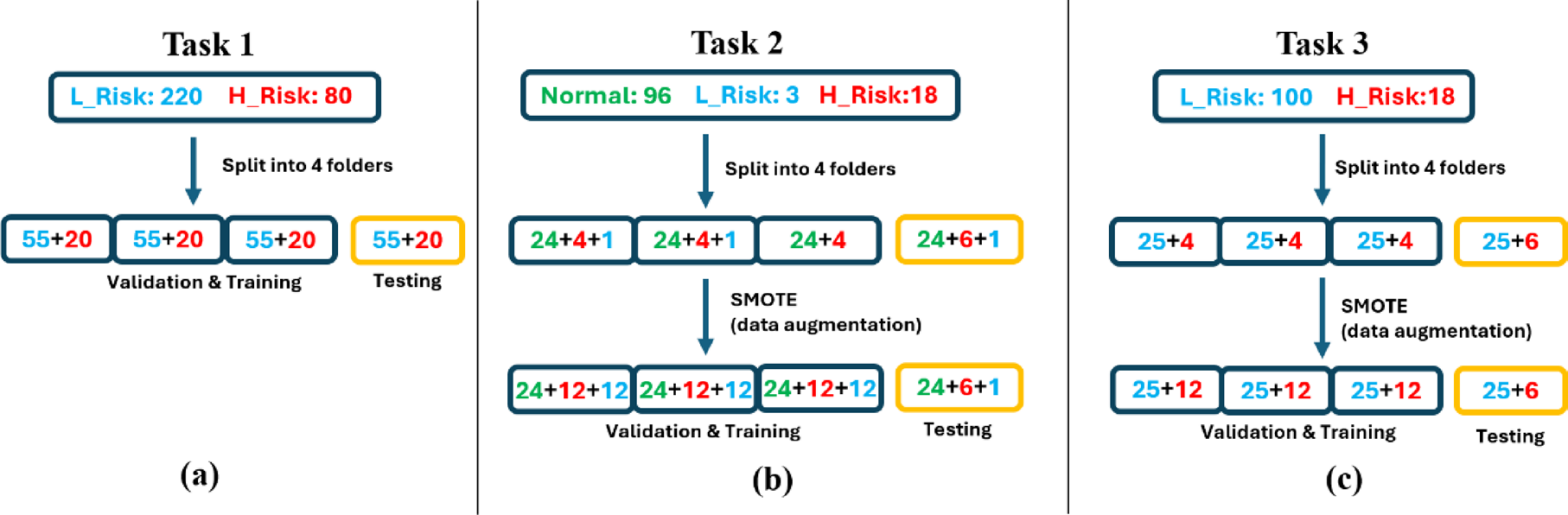
Data separation and augmentation pipeline of each trial for Task 1/2/3.

**Task 1: Binary classification (low-risk/high-risk) applied to pig mucosal data**.

A total of 220 EIS spectra from healthy pig mucosa (VT & FM) data were categorized as **low-risk** and the 80 readings obtained from acetic acid-treated mucosa were categorized as high-risk. K-fold cross validation (K = 3) was employed to determine the model structure and configure hyperparameters related to class weights and L2 regularization. Within each fold of every trial, there were 55 instances of low-risk data and 20 instances of high-risk data (Fig. 5a). Overall, 100 trials (where different splits of the training, validation and testing data were applied) were conducted from which the means of the 100 validation performance evaluation results (in terms of AUC/ACC) were gathered and utilized to select the best model structure parameters. Subsequently, the chosen models would be trained using both the training and validation datasets and tested on the test fold.

**Task 2: Three group classification (Normal/Low/High-risk) applied to human data**.

The dataset comprised 96 normal, 3 low-risk, and 18 high-risk EIS spectra from human subjects. K-fold cross validation (K = 3) was utilized to determine the optimal combination of the 5 hyperparameters for the model. As a data augmentation technique during data separation, SMOTE (Synthetic Minority Over-sampling Technique)^29^ was employed to increase the quantity of low-risk and high-risk instances in the training dataset. Each trial records 3 validation outcomes (trained on 2-folds and validated on 1-fold) as shown in Fig. 5b. Overall, the training was conducted 100 times across 100 trials, and the average of the 100 validation performance evaluation results (in terms of AUC/ACC) were collected to determine the best model configuration using multi-class AUC^30^. Subsequently, the selected models underwent training using both the training and validation datasets, followed by testing on the test data.

**Task 3: Binary classification (Low/High-risk) applied to human data**.

In Task 2, there were only 3 low-risk cases, which were insufficient for CNN models to learn meaningful features. To address this issue and create a more balanced dataset, in Task 3, the low-risk cases were combined with the 97 normal cases to form a new composite low-risk class. This approach balanced the class distribution and provided the CNN model with a more substantial number of samples in the low-risk class. Like Task 2, SMOTE was used to augment the size of the training and validation dataset as shown in Fig. 5c. The data separation was again repeated 100 times to ensure a comprehensive utilization of the entire dataset.

In all experiments, data were split such that no data from the same subject appeared in both training and testing sets. This was enforced during each of the 100 repeated random splits in Task 2 and Task 3. However, due to the small number of high-risk cases, we could not strictly enforce subject-level separation within cross-validation folds (training/validation splits). SMOTE was applied only within the training folds to address class imbalance.

### Model training and model performance analysis

During the phase of model training for these tasks, different models with distinct structures were trained using the training and validation data. The performances of different models were compared using the receiver operating characteristic (ROC) curves area under the curve (AUC). The difference between the performances was evaluated statistically using Wilcoxon signed-rank test^31,32^ with the significance level α = 0.05. The structures of the top 2–4 best-performing models according to the criterion of cross-validation were selected. Subsequently, the training and validation data are merged for training the models with the best structures and the performances of the trained models were evaluated on the test data using the metrics of AUC or accuracy (ACC) defined as follows:

3 $$\:\begin{array}{c}ACC=\frac{TP+TN}{TP+FP+FN+TN}\end{array}$$

where TP: Number of true positive cases; TN: Number of true negative cases; FP: Number of false positive cases; FN: Number of false negative cases.

### Threshold adjustment algorithms

**General Principle: Yudens’ rule**

When a machine learning model is applied to perform a classification task on testing data, an optimal threshold derived from the training data Receiver Operating Characteristic (ROC) curve is normally used, which is crucial for the classification performance of the machine learning model. The determination of the threshold using the training data ROC is often based on the so-called Yudens’ rule^33^, which was derived using the concepts of sensitivity and specificity defined by

4 $$\:\begin{array}{c}Specificity=\frac{TN}{FP+TN}\end{array}$$

5 $$\:\begin{array}{c}\:Sensitivity=\frac{TP}{TP+FN}\end{array}$$

and can be described as follows:

Yudens’ rule is a measure used to assess the effectiveness of a binary classification system. It calculates the optimal balance between sensitivity (true positive rate) and specificity (true negative rate) by the formula:

6 $$\:\begin{array}{c}J=Sensitivity+Specificity-1\end{array}$$

A higher score of J indicates a better classifier performance, with a range from 0 (no better than random) to 1 (perfect classification). It helps in choosing the best threshold and comparing different classifiers effectively. However, in many medical diagnoses, the Yudens’ rule may not be always followed for the threshold determination. Instead, the threshold is determined according to the specificity or sensitivity required for individual medical diagnosis problems. For example, epidemic screenings often need a higher specificity while in cancer diagnoses a higher sensitivity is often required^34^. Consequently, a threshold most appropriate for the diagnosis problem to be addressed should be used. Following this principle, in the present study, the Yudens’ rule is applied to determine the threshold for Task 1 while the following threshold adjustment algorithms are proposed to determine the threshold for Tasks 2 and 3, respectively.

**Threshold adjustment algorithm for Task 2**.

To perform the diagnostic Task 2, given the scores produced by the classification model trained for Task 2 are z_1_, z_2_, and z_3_ representing the probabilities of normal (Category 1), low risk (Category 2), and high risk (Category 3), respectively, the conventional approach to making the diagnosis decision is to choose Category i^*^ such that

7 $$\:\begin{array}{c}{i}^{*}=\underset{i\in\:\left\{\text{1,2},3\right\}}{arg}{max}\left\{{z}_{1},{z}_{2},{z}_{3}\right\}\end{array}$$.

In the present study, however, the requirement for a reasonably high sensitivity is considered as this is a cancer diagnosis problem. For this purpose, the following algorithm is proposed to make the diagnostic decision:

If z_3_ ≥ T* then the classification outcome is “high risk”, otherwise if z_1_ ≥ z_2_, the classification outcome is “normal” but if z_2_ > _Z1_, the classification is “low risk”.

Here, T* is the threshold to be determined for Task 2. To determine T*, the sensitivity and specificity of the finally trained classifier for Task 2 under threshold T* are evaluated with T* decreasing from 0.5 to 0.15 until the trained classifier with threshold T* can achieve a balanced performance in terms of sensitivities of classification over different classes.

Finally, threshold T* thus determined is applied to the test set to assess the model’s predictive capability in practical scenarios, ensuring it aligns with clinical needs.

**Threshold adjustment algorithm for Task 3**.

To perform the diagnostic Task 3, the requirement for a reasonably high sensitivity is also considered. Because this is a two-class classification problem, the Yudens’ rule based optimal threshold is determined first and denoted as T_Y_. Then, the sensitivity and specificity of the finally trained classifier for Task 3 under threshold α T_Y_ are evaluated with α decreasing from 1 to 0.1 until α = α* such that the trained classifier with threshold α* T_Y_ can achieve both a satisfactory sensitivity and an acceptable specificity. Finally, the threshold α*T_Y_ is applied to the test set to assess the model’s predictive capability in practical scenarios, ensuring it aligns with clinical needs.

## Results

### Deep learning-based analysis of Porcine mucosal EIS data for abnormal diagnosis

**Fig. 6:**
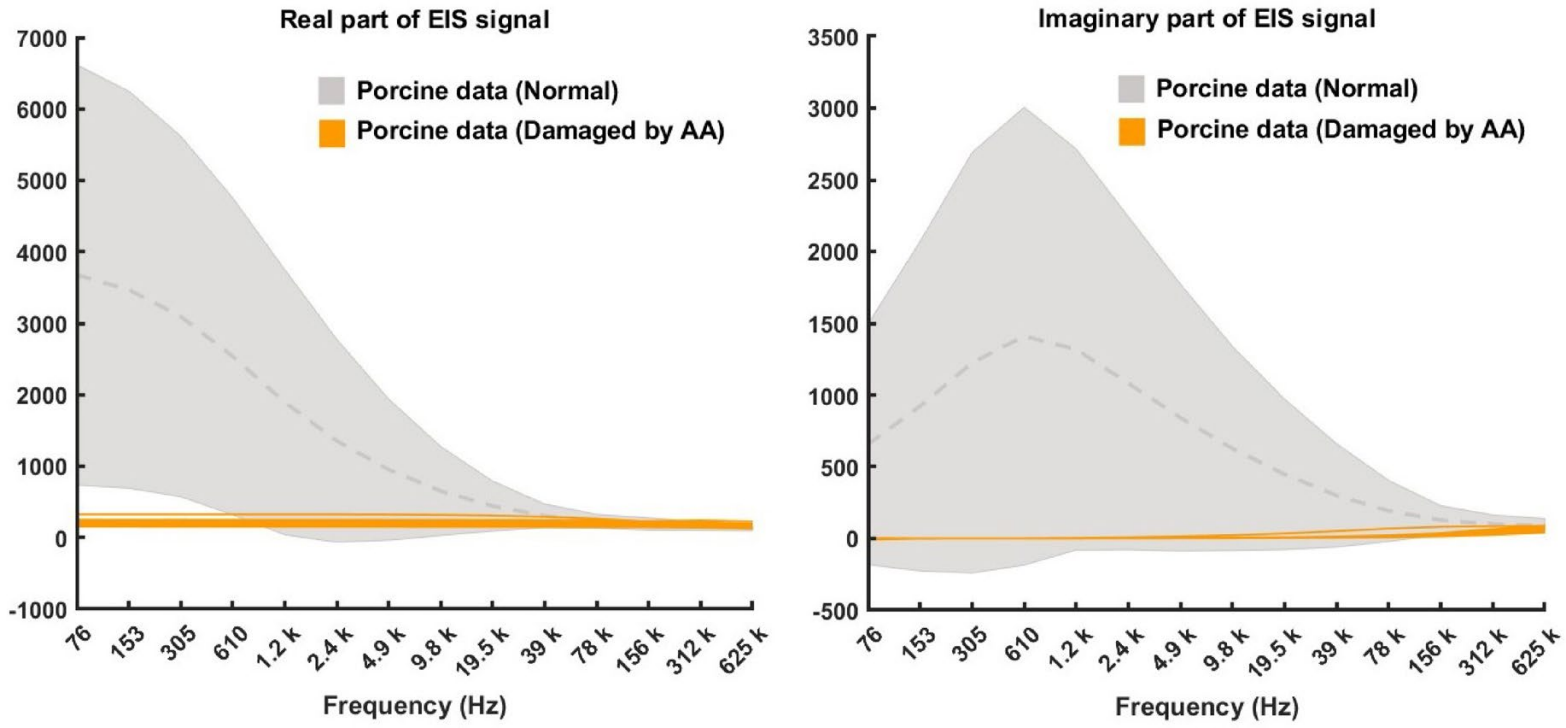
Comparison of the EIS spectra of the pig heads without/with chemical treatment. The grey area represents the distribution of normal pig samples, with the dashed line indicating the mean and its bounds set by the standard deviation. The orange line represents samples damaged by acetic acid.

The differences between the spectra derived from pig heads treated with acetic acid and the normal data shown in Fig. 6 were similar to the differences between the data collected from the normal human mouth and the data from high-risk patients, which can be found in^19^.

**Table 2:** Validation AUC performance of task 1. Each row denotes a model structure using the notation model_xl_yc_z (x = number of hidden layers, y = number of convolutional layers, z = neurons per hidden layer). Each column heading (e.g., 1:1 0.01) represents a training condition, where 1:1 indicates class weights used in the loss function, and 0.01 is the value of the L2 regularization parameter λ.

Valid_AUC	Mean ±SD [%]			
	1:1 0.01	1:1 0.1	0.1:1 0.01	0.1:1 0.1
Model_2 l_0c_1	0.931 ±0.018	0.929 ±0.014	0.932 ±0.012	0.931 ±0.018
Model_2 l_0c_2	0.926 ±0.019	0.924 ±0.017	0.928 ±0.015	0.934 ±0.013
Model_2 l_2c_1	0.956 ±0.021	**0.963 ±0.016**	0.958 ±0.021	0.962 ±0.017
Model_2 l_2c_2	0.956 ±0.021	**0.964 ±0.017**	0.958 ±0.016	**0.963 ±0.016**
Model_3 l_0c_1	0.928 ±0.022	0.930 ±0.016	0.929 ±0.019	0.933 ±0.015
Model_3 l_0c_2	0.920 ±0.023	0.928 ±0.014	0.928 ±0.014	0.931 ±0.014
Model_3 l_2c_1	0.959 ±0.021	0.958 ±0.021	0.958 ±0.018	0.958 ±0.018
Model_3 l_2c_2	0.958 ±0.025	**0.964 ±0.021**	0.958 ±0.021	0.957 ±0.018

Table 2 shows the results of model validation of Task 1. It can be observed that the models utilizing CNN with convolutional layers have secured a top-4 position and are marked in bold in Table 2, having a mean of AUC from 0.963 ± 0.016 to 0.964 ± 0.021. These models outperformed those lacking convolutional layers but employing the same training strategy ($\:p<0.001$). The class weight parameter did not significantly affect the results, indicating the independence of the results from class imbalance.

When employing different hyper-parameter settings, CNN models demonstrated a 3–4% enhancement in AUC compared to basic neural models lacking convolutional operations ($\:p<0.001$). In NN without convolution, the difference between two-layer and three-layer models was not prominent. Regarding CNN models, an increase in λ from 0.01 to 0.1 resulted in a gain of approximately 4–7% in AUC ($\:p<0.05$).

During the testing phase, as shown in Supplementary Table S2, model_2 l_2c_2 (with 0.1:1 0.1, test AUC: 0.977 ± 0.015, $\:p$ = 0.007–0.015) and model_3 l_2c_2 (with 1:1 0.1, test AUC: 0.979 ± 0.015, $\:p$ = 0.043–0.045) secured the top 2 positions.

In a binary classification, the AUC of ROC shows the overall performance of the classifier, but a specific classification result needs to be determined using a threshold that is determined from ROC using either Yuden’s rule or other algorithms to achieve a balanced performance in terms of sensitivity and specificity. In principle, the threshold is determined from the ROC of the training data and then applied to testing data to perform the classification for unseen scenarios. In Task 1, this principle was followed with Yuden’s rule being applied to determine the optimal threshold from the ROC of the training data for each of the 100 trials.

**Fig. 7:**
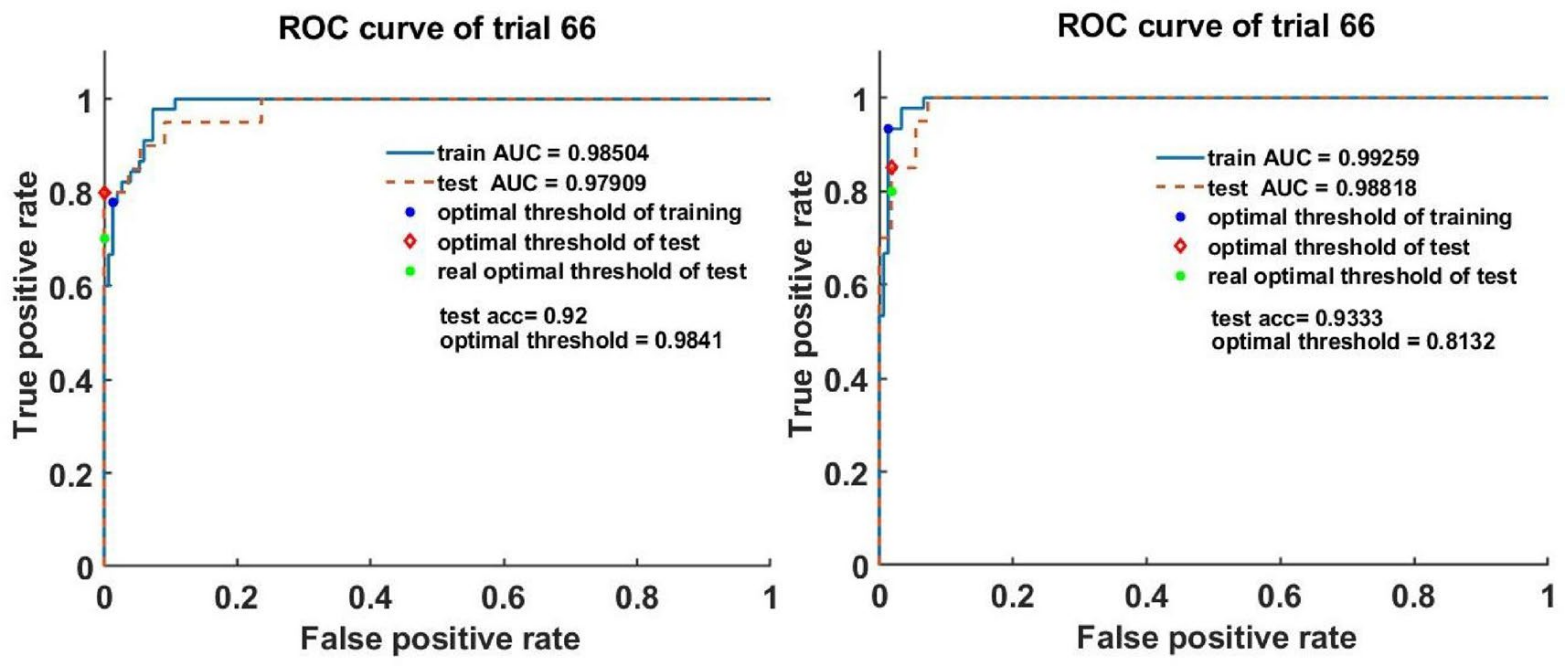
The ROC curves of model_2 l_2c_2 under 0.1:1 0.1(left) and model_3 l_2c_2 under 1:1,0.1 (right) from 66 th of the 100 trials in total.

Figure 7 shows the ROC from both the training and testing data of model_2 l_2c_2 under 0.1:1,0.1 and model_3 l_2c_2 under 1:1,0.1, respectively, which were obtained from one of the 100 trials (Trial 66). It can be observed from Fig. 7 that the training and testing data ROC are not the same but similar. When applying the optimal threshold from training data to the testing data, the classifier does not perform optimally because there is a difference between the optimal threshold with training data ROC and the optimal threshold with testing data ROC.

Considering this and to more comprehensively evaluate the performance of the two top models, for each trial, the optimal threshold is first determined from the ROC of the training data. Then, the performance indices (ACC, sensitivity, and specificity) of the models when applied to the testing data using the training data produced threshold is evaluated. After that, the average of these performance indices over the 100 trials were evaluated and are shown in Supplementary Table S3 and S4.

It can be observed for the results in Supplementary Table S3 and S4 that although there exists a difference between the optimal thresholds with training and testing data, the direct application of the optimal threshold with training data to the analysis of testing data as can literally be implemented in practice can still achieve a satisfactory diagnosis accuracy (Accuracy: 91.2–92.2%, ± 0.035 ($\:p<0.05$); Specificity: around 0,95; and Sensitivity: 0.81–0.84 ($\:p<0.05$)). This demonstrates that CNNs can effectively and robustly be used to discriminate between healthy and damaged pig mucosa. The same principle was next applied to investigate the performance of CNN when applied to human clinical data for oral cancer diagnosis.

### Application of the CNN models to human oral EIS data for classification of three clinical conditions

**Table 3:** Validation AUC performance of task 2. Each row denotes a model structure using the notation model_xl_yc_z (x = number of hidden layers, y = number of convolutional layers, z = neurons per hidden layer). Each column heading (e.g., 1:1 0.01) represents a training condition, where 1:1 indicates class weights used in the loss function, and 0.01 is the value of the L2 regularization parameter λ.

	Mean ± SD [%]			
	1:1:1 0.01	1:1:1 0.1	0.1:1:1 0.01	0.1:1:1 0.1
Model_2 l_0c_1	0.922 ±0.044	0.930 ±0.030	0.941 ±0.030	0.935 ±0.029
Model_2 l_0c_2	0.924 ±0.034	0.921 ±0.029	0.929 ±0.030	0.930 ±0.029
Model_2 l_2c_1	0.953 ±0.020	0.953 ±0.020	**0.958 ±0.020**	**0.961 ±0.018**
Model_2 l_2c_2	0.953 ±0.025	0.953 ±0.024	**0.957 ±0.022**	**0.960 ±0.019**
Model_3 l_0c_1	0.940 ±0.030	0.935 ±0.029	0.948 ±0.025	0.944 ±0.023
Model_3 l_0c_2	0.930 ±0.029	0.921 ±0.031	0.940 ±0.027	0.933 ±0.022
Model_3 l_2c_1	0.949 ±0.024	0.944 ±0.027	0.956 ±0.021	0.954 ±0.019
Model_3 l_2c_2	0.949 ±0.029	0.944 ±0.029	0.956 ±0.024	0.955 ±0.022

The multi-class AUC is usually calculated by converting the problem into multiple binary classification problems. A common approach is to use the ‘single category versus other categories’ approach, where each category against a combination of all other categories forms a binary classification problem. Then, for each binary classification problem, an ROC curve can be calculated, and the corresponding AUC can be calculated. Finally, the average of the AUCs over all categories produces the multi-class AUC. Therefore, there are K classes, the calculation for the multi-class AUC is as follows:

For each class $\:i$, the remaining K-1 classes are combined to form a binary classification problem. Thus, there are K binary classification problems. For the i^th^ of these binary classification problems, the ROC curve and the corresponding AUC is calculated, producing AUC_i_. The AUCs of all these binary classification problems is then averaged to determine an overall AUC as

8 $$\:\begin{array}{c}MultiClas{s}_{AUC}=\:\frac{1}{K}\sum\:_{i=1}^{K}{AUC}_{i}\end{array}\:$$.

Based on the multi-class classification AUC defined above, the validation AUC for each candidate NN structure was evaluated and the results are as shown in Table 3. Under the same training strategy, the models with a convolutional layer show higher validation AUC ($\:p<0.05$). The two-layer CNN model with convolutional layers achieved a top-4 position among all the models during validation (AUC around 0.96 ± 0.02). Additionally, during training, the ‘normal’ class with the top ranked models had a lower weight (weight=0.1 in the loss function, $\:p<0.05$). When trained with different values of λ for L2 regularization, the models showed non-significant impact of L2 regularization ($\:p>0.05$).

Under different training strategies involving variations in class weights in the loss function and λ values, the two-layer models with convolutional layers exhibited a significant improvement of 1–3% in AUC compared to models without convolutional layers ($\:p<0.05$). For three-layer models, this improvement ranged from 1 to 2% ($\:p<0.05$). When there were no convolutional layers, increasing the number of neurons in the hidden layers from 125 to 250 led to a significant decrease of 0.5–1% in AUC for all models ($\:p<0.01$, except 3-layers model without convolutional layer under 0.1:1:1 0.1, $\:p$ = 0.056). After adding convolutional layers, if class weights were kept consistent (1:1:1) and λ = 0.1, the three-layer models experienced a significant decrease in AUC of approximately 0.5–1% compared with two-layer models ($\:p<0.05$). After adjusting the class weights, with the weight of the normal class decreasing from 1 to 0.1, each model’s AUC showed an improvement of approximately 0.5–1.1% ($\:p<0.05$; except model_2 l_0c_1 under λ = 0.01, $\:p$ = 0.066 or under λ = 0.1, $\:p$ = 0.096; model_2 l_0c_2 under λ = 0.01, $\:p$ = 0.283).

**Table 4:** Testing AUC and ACC results of task 2.

	Mean ± SD [%]			
	0.1:1:1 0.01		0.1:1:1 0.1	
	Test_AUC	Test_ACC	Test_AUC	Test_ACC
Model_2 l_2c_1	0.921 ±0.068	0.861 ±0.060	0.915 ±0.071	0.855 ±0.066
Model_2 l_2c_2	0.918 ±0.068	0.877 ±0.051	0.911 ±0.067	0.844 ±0.071

In terms of testing results, as shown in Table 4, Model_2 l_2c_2 achieved the best accuracy (0.877 ± 0.051, $\:p<0.05$). Under the training strategy with λ = 0.01, the models achieved higher test AUC scores than under the strategy of λ = 0.1.

Since there was only one low-risk case in the test data set of each trial, the test data results of all 100 trials of each model were pooled for further analysis. The results are shown in Table 5.

**Table 5:** Distribution of classification results with four top NN models under conventional threshold selection. (m1: Model_2 l_2c_1 0.1:1:1 0.01; m2: Model_2 l_2c_2 0.1:1:1 0.01; m3: Model_2 l_2c_1 0.1:1:1 0.1; m4: Model_2 l_2c_2 0.1:1:1 0.1).

	Normal	Low-risk	High-risk
	Classification distribution for **normal** cases in all trails		
m1	0.902	0.034	0.064
m2	0.929	0.024	0.048
m3	0.888	0.036	0.076
m4	0.880	0.034	0.087
	Classification distribution for** low-risk **cases in all trails		
m1	0.170	0.830	0.000
m2	0.100	0.870	0.030
m3	0.130	0.850	0.020
m4	0.120	0.880	0.000
	Classification distribution fo**r high-risk **cases in all trails		
m1	0.285	0.013	0.702
m2	0.305	0.020	0.673
m3	0.272	0.005	0.723
m4	0.277	0.027	0.697

From the results in Table 5, it is known that, for the normal sample, the cases of misclassification are less concentrated on the high-risk side. For low-risk samples, the results of misclassification are almost always concentrated on normal cases. These results are all acceptable because normal and low risk samples are not clinically contentious with the corresponding mucosal tissues being likely healthy and not in danger of progressing to cancer. However, for high-risk samples, nearly 30% of the samples were misclassified as normal, meaning a sensitivity of high-risk = 0.7. Clinically, this scenario is not appropriate because a significant percentage of high-risk OPMD lesion or even OSCC cases could be mis-diagnosed as normal. The fundamental reason with this issue is that the NN models use the inherent highest probability principle to perform the classification tasks.

To resolve this problem, in the present study, the threshold adjustment method for Task 2 as described in Sect. "Threshold adjustment algorithms" is proposed and applied in here. The basic idea is to classify a test case into high-risk category provided the NN model produced score/probability of high risk is larger than a predefined threshold T*. Otherwise, the result class will belong to the class with the highest probability. In addition, the training data were used to determine T* to achieve a balanced performance in terms of sensitivities of classification over different classes.

**Fig. 8:**
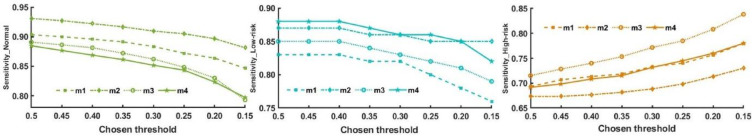
The classification sensitivity for normal (left), low risk (middle) and high risk (right) classes under different thresholds. Figure 8 shows the variations of classification sensitivities for normal, low risk, and high risk classes achieved by the four top models (m1-m4) on training data with the decrease of the high-risk threshold T* from 0.5 to 0.15.

It can be found that the reduction of threshold from 0.5 to 0.15 results in a decline in sensitivity for normal and low-risk cases but an increase in sensitivity for high-risk case. It can also be observed from Fig. 8 that, with the threshold ranging between 0.2 and 0.25, the sensitivity for high-risk case becomes much better than before (when using highest probability principle) while the sensitivities for other two cases (specificity for non-high-risk case) can still maintain an acceptable range. Consequently, T*=0.2 is used as the threshold for the case of high-risk for Task 2. Table 6 shows the sensitivity results for each case when T*=0.2 is used as the high-risk threshold while Table 7 provides a summary of the distribution of classification results with the four top NN models under this threshold. A comparison of the results in Table 7 with that in Table 5 show that, after the high-risk threshold was selected as T*=0.2, each model can achieve an increased sensitivity of 5.5–8.5 more percentage points for high-risk case and a 5–8.5% point decrease in misassigning high-risk cases to normal. This demonstrates the effectiveness of the proposed threshold adjustment algorithm for Task 2.

**Table 6:** Sensitivity analysis for different cases when high-risk case threshold = 0.2.

Model	Sensitivity_Normal	Sensitivity_Low-risk	Sensitivity_High-risk
m1	0.864	0.760	0.780
m2	0.898	0.850	0.713
m3	0.830	0.810	0.808
m4	0.823	0.850	0.760

**Table 7:** Distribution of classification results with four top NN models under the threshold selection proposed for task 2.

	Normal	Low-risk	High-risk
	Classification distribution for **normal** cases in all trails		
m1	0.864	0.031	0.105
m2	0.897	0.022	0.081
m3	0.830	0.033	0.137
m4	0.823	0.032	0.145
	Classification distribution for **low-risk** cases in all trails		
m1	0.170	0.780	0.050
m2	0.100	0.850	0.050
m3	0.130	0.810	0.060
m4	0.120	0.850	0.030
	Classification distribution for **high-risk** cases in all trails		
m1	0.235	0.008	0.757
m2	0.267	0.020	0.713
m3	0.187	0.005	0.808
m4	0.217	0.023	0.760

### Application of the CNN models to human oral EIS data for classification of two clinical conditions

**Table 8:** Validation AUC performance of task 3. Each row denotes a model structure using the notation model_xl_yc_z (x = number of hidden layers, y = number of convolutional layers, z = neurons per hidden layer). Each column heading (e.g., 1:1 0.01) represents a training condition, where 1:1 indicates class weights used in the loss function, and 0.01 is the value of the L2 regularization parameter λ.

	Mean ±SD [%]			
	1:1 0.01	1:1 0.1	0.1:1 0.01	0.1:1 0.1
Model_2 l_0c_1	0.922 ±0.044	0.918 ±0.044	0.919 ±0.046	0.905 ±0.051
Model_2 l_0c_2	0.919 ±0.035	0.917 ±0.040	0.911 ±0.049	0.898 ±0.048
Model_2 l_2c_1	0.937 ±0.039	**0.942 ±0.034**	0.934 ±0.044	0.937 ±0.037
Model_2 l_2c_2	0.934 ±0.033	**0.940 ±0.033**	**0.937 ±0.032**	**0.939 ±0.034**
Model_3 l_0c_1	0.921 ±0.039	0.923 ±0.039	0.926 ±0.038	0.924 ±0.038
Model_3 l_0c_2	0.916 ±0.043	0.906 ±0.045	0.920 ±0.037	0.910 ±0.045
Model_3 l_2c_1	0.929 ±0.034	0.932 ±0.037	0.934 ±0.034	0.935 ±0.037
Model_3 l_2c_2	0.925 ±0.044	0.930 ±0.045	0.930 ±0.039	0.934 ±0.040

For the two-class classification task, the validation AUC for each candidate NN structure was evaluated and the results are as shown in Table 8. The CNN models outperformed the models without a convolutional layer with significance ($\:p<0.05$; except 3-layers model with 125 neurons in each hidden layer, $\:p$ = 0.05–0.08), leading to a rise in AUC by 1–4%. Not all 2-layer CNN models show significant improvement over 3-layer CNN models in the same training mode, e.g., Model_2 l_2c_1 under 1:1 0.1, $\:p$ = 0.005; Model_2 l_2c_2 under 1:1 0.1, $\:p$ = 0.061; Model_2 l_2c_2 under 0.1:1 0.01, $\:p$ = 0.017; Model_2 l_2c_2 under 0.1:1 0.1, $\:p$ = 0.102. Among all CNN models utilizing different class weight pairs and λ values, four 2-layer CNN models marked in red text in Supplementary Table S5 with the top 4 AUC were selected in the next testing period.

In terms of testing results, as shown in Supplementary Table S5 and S6, Model_2 l_2c_2 achieved the top 2 places ($\:p<0.05$), test AUC: 0.907±0.091 under 1:1 0.1; 0.903 ± 0.092 under 0.1:1 0.1. The top 2 models achieved a testing accuracy of approximately 90.4 ± 0.04% when the Yuden method based threshold obtained from the training data is applied, indicating robust results.

Due to few high-risk data (*n* = 6) in the test data in one trial, the testing results from all trials of one model are pooled for the prediction analysis as shown in Supplementary Table S7. For both models, around 97–98% of the low-risk cases are classified correctly, meaning that trained models are sensitive to normal data. Conversely, both models exhibited poorer performance in sensitivity, misclassifying around 36–41% of the high-risk test data as normal. The results exhibit a similar trend as in Task 2 when the conventional thresholding approach is applied, suggesting the potential for this approach to serve as a broad screening method for oral cancer diagnosis when directly employing the optimal threshold determined using the Yuden method from training ROC curves.

However, it is crucial to acknowledge that oral cancer, as a high-risk disease, could lead to significant psychological, physiological, and economic implications for patients who might be missed during screening. Therefore, it is essential to achieve higher sensitivity for this diagnosis problem. For this purpose, instead of Yuden’s method, the threshold determination algorithm for Task 3 as introduced in Sect. 2.7 is applied.

**Fig. 9:**
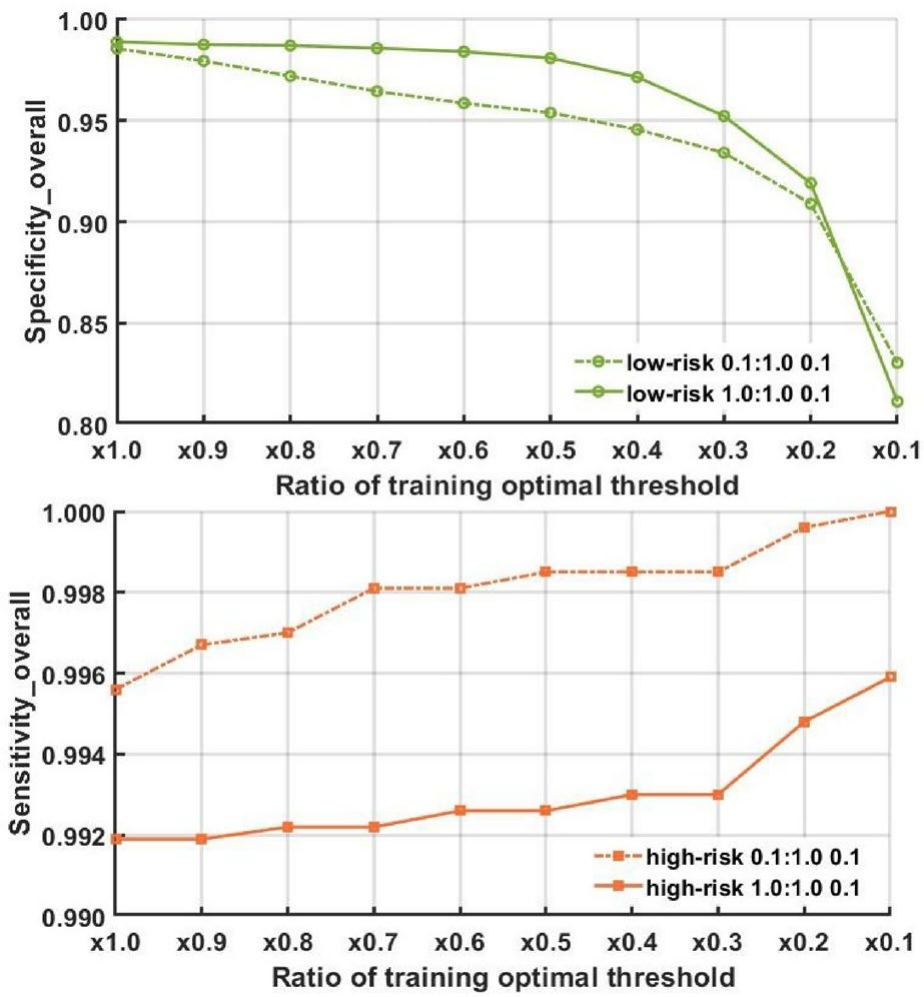
Specificity and sensitivity performance with different ratio of optimal threshold in training dataset.

Figure 9 shows the variation of sensitivity and specificity of the two top models under threshold α _*_T_Y_ with the decrease of α from 1 to 0.1 where it can be observed that when α = 0.3, the sensitivity of Model_2 l_2c_2 under 0.1:1.0:0.1 reaches a value over 0.998, and the specificity is around 0.93–0.95, which is also very good.

**Fig. 10:**
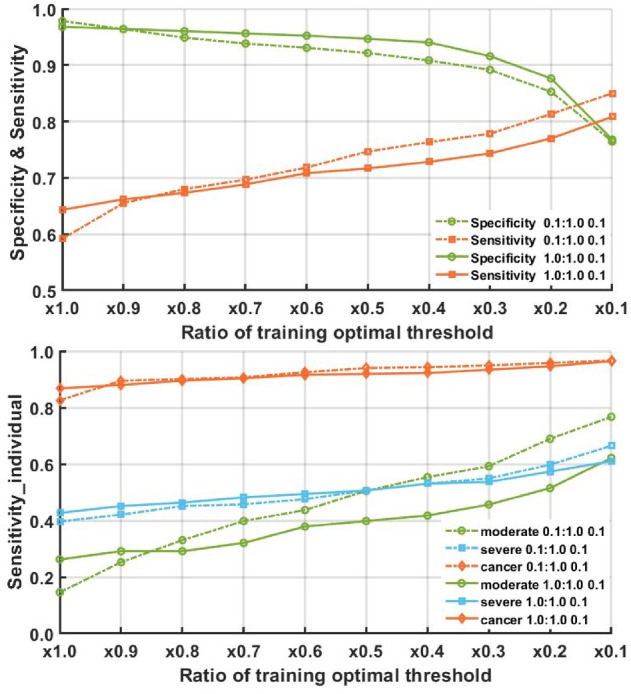
The specificity and sensitivity of testing results under different thresholds (top) and individual sensitivity analysis (bottom) for specific cases (cancer/severe dysplasia/moderate dysplasia) among high-risk cases. The sensitivity analysis (bottom) is based on the results of combining all high-risk data from 100 trials (600 cases in total). The figure is drawn by calculating the classification accuracy of each class under different thresholds.

In testing results, when choosing different thresholds, the overall sensitivity is approximately 0.76 (0.743 under 1:1 0.1, 0.778 under 0.1:1 0.1) when ratio = 0.3 as shown in Fig. 10. In high-risk data, with an optimal threshold ratio of 0.3, the sensitivity for cancer cases could reach 0.94 (0.934 under 1:1 0.1, and 0.949 under 0.1:1 0.1). Furthermore, a noticeable improvement is observed in moderate dysplasia data, increasing from 0.146 (ratio = 1.0) to 0.592 (ratio = 0.3). The best ratio (= 0.3) is selected based on the training ROC results, and subsequently, the ratio is adjusted from 0.2 to 0.4 to determine if it remains optimal in the testing results.

For the case of each model as shown in Table 9, the result obtained with ratio = 0.3 outperforms the others, indicating that the method of modifying the threshold using training results and applying the modified threshold in practice is a feasible approach in future applications.

**Table 9:** Performance comparison under different thresholds.

Model	Specificity	Sensitivity
Testing prediction results (Threshold Ratio = 0.3)		
0.1:1.0 0.1	0.892	0.778
1.0:1.0 0.1	0.916	0.743
Testing prediction results (Threshold Ratio = 0.2)		
0.1:1.0 0.1	0.853	0.813
1.0:1.0 0.1	0.876	0.770
Testing prediction results (Threshold Ratio = 0.4)		
0.1:1.0 0.1	0.908	0.763
1.0:1.0 0.1	0.940	0.728

## Discussion

Advancements in non-invasive tissue monitoring devices are required to meet some of the limitations of the current ‘gold standard’ surgical biopsy and histopathological assessment of OMPD, where concerns over consistency in pathological diagnosis still exist^35,36^. Such non-invasive devices could be deployed as clinical assessment aids for the early detection of precancerous lesions and longitudinal monitoring of suspicious OPMD. Visualisation of suspicious oral lesions using either dyes or auto-fluorescence upon blue light illumination is an option, but there is significant debate about the clinical effectiveness of these techniques^37^. Electrical impedance spectroscopy (EIS) has previously been applied in cervical^18^, breast^15^, and skin cancer^14^ diagnostics due to its ability to detect changes in the structure and composition of normal compared to diseased tissue in a non-invasive manner. Its low cost, real-time feedback and portability make it an appealing adjunctive tool for early screening. However, EIS is sensitive to contact pressure, probe positioning and tissue hydration, which can introduce signal variability and require careful protocol standardization. Moreover, although EIS may significantly aid in the early detection and screening of suspicious oral lesions, it cannot replace surgical biopsy and histopathological examination for a definitive diagnosis.

In comparison to technologies such as optical coherence tomography (OCT) and in vivo confocal microscopy, which offer higher spatial resolution, EIS provides greater depth of field and is less dependent on optical contrast. OCT and confocal systems require more sophisticated hardware and trained operators, whereas EIS could be deployed more easily in low-resource settings. There is also the possibility that these technologies could be combined to provide enhanced non-invasive diagnostics. This highlights its practicality in wide screening contexts and may further confirm the need of the application of machine learning which can systematically combine the results of different screening techniques, providing a better informed diagnosis.

This study explores the potential of the application of CNN-based deep learning in conjunction with EIS as a non-invasive method for the early detection of oral lesions that are either at high-risk of progressing to cancer or are already cancerous.

The use of both WHO grading and binary risk stratification reflects real-world diagnostic uncertainty and clinical needs. While the WHO system offers detailed histopathological granularity (e.g., mild, moderate, severe dysplasia), its application in this study was limited by the relatively small dataset, which would result in insufficient sample sizes for each subclass in DL model training. Therefore, the primary analysis focused on the binary classification system, which simplified the model training process and better supported the development of a clinically relevant screening tool. Binary stratification also aligns more closely with real-world decision pathways (e.g., surveillance vs. biopsy), making it a practical choice for early-stage lesion identification. We also acknowledge that with the limited number of lesion cases, further subdivision beyond binary categories, such as finer grading of dysplasia, may lack statistical power and robustness. Future studies with larger and more balanced datasets will be needed to explore meaningful subclassifications.

Overall, three tasks were completed. In Task 1, we explored the use of pig mucosal tissue that is histologically similar to human oral mucosa^24^ to test the feasibility of the proposed EIS/CNN approach. Given the moderate balance between the two classes (normal and high-risk) in pig head samples, the results showed no significant deviation in the model’s specificity (0.949–0.952) and sensitivity (0.812–0.840), both higher than 80%. Table 2 showed that in the validation set, the neural network with additional convolutional layers exhibited a roughly 3% increase in AUC compared to the standard neural network ($\:p<0.05$). This implies that the convolutional layers utilized in this study were capable of extracting additional non-linear features from the data, which may improve feature representation and support learning in this context. In Task 2 (three classes’ classification task on human oral mucosa), when conventional Yuden technique was used to determine the optimal threshold, around 30% of samples that should have been categorized as high-risk were misclassified as normal. This can be due to a significant imbalance of data in normal and high-risk cases. To address the misclassification problem, a threshold adjustment method was proposed to satisfy the need of cancer diagnosis, which increases the sensitivity from around 0.7 (0.673–0.723) to around 0.76 (0.713–0.808). Using the same threshold adjustment approach in Taks 3 (two classes’ classification task on human oral mucosa), the sensitivity of both top-2 models was increased from 0.592 (0.643) to 0.778 (0.743). Therefore, the proposed threshold adjustment could, to some extent, compensate for the issue of low sensitivity caused by an insufficient and imbalanced dataset. It is worth emphasizing that because of the implication of the outcome of cancer diagnosis, clinicians may opt to prioritize relatively higher sensitivity in the screening process. This proposed adjustment of the threshold may help address this practical need and could contribute to the development of models tailored to specific screening requirements, maintaining a balance between sensitivity, cost efficiency, and the nature of the disease being addressed.

From these tasks, it was also observed that models with one-dimensional convolutional layers exhibited significantly better classification accuracy under the same training hyperparameter settings ($\:p<0.05$). This improvement can be attributed to one-dimensional convolutional layers acting as filters. Through convolutional operations, they enable interactions between impedance values of adjacent data points, allowing for the extraction of deeper-level or high-dimensional information^38^. However, augmenting the quantity of hidden layers did not notably enhance model accuracy, with the most optimal models predominantly featuring two hidden layers. While deeper architectures theoretically possess a greater potential for function approximation, they may introduce overfitting concerns and elevate training complexity, leading to more challenging convergence^39^. Consequently, for the comparatively simple dataset in the present study, augmenting the number of hidden layers did not yield significant advantages. Similarly, the quantity of neurons in the hidden layers should be tailored to the dataset’s complexity. Overall, the integration of CNNs with EIS allows for automated feature extraction and classification, reducing reliance on subjective interpretation. CNNs can identify non-linear patterns within high-dimensional data, improving diagnostic consistency. However, these models are often seen as ‘black boxes’ which may limit clinical adoption unless interpretability methods or risk-based decision thresholds are incorporated. Our study addresses part of that challenge through the proposed threshold adjustment strategies for clinical relevance.

This study has several limitations. First, the relatively small number of lesion cases restricts the generalizability of the findings and may limit the model’s performance in wide populations. Second, the imbalance in class distribution poses a risk of model bias, although strategies such as weighted loss functions were employed. Additionally, although care was taken to prevent subject overlap and data leakage between training and testing sets, subject-level separation could not be guaranteed within the cross-validation folds during training due to the limited number of high-risk cases. This may have introduced some degree of data leakage. However, we addressed this issue by repeating the entire process 100 times per model with new random splits to improve robustness and reduce bias. The evaluation of the final best model is also based on the testing set to ensure that no data leakage problem has been received. Lastly, the current model was trained and validated on data from a limited clinical record, and further external validation across multiple groups is essential to confirm its robustness.

Future work will focus on expanding the dataset size to improve the representativeness and statistical analysis. Additionally, incorporating interpretability methods such as attention mechanisms or using prior knowledge may enhance clinical trust and transparency. Efforts will also be made to evaluate the integration of EIS-CNN systems into real-time diagnostic workflows and to compare performance with existing standard-of-care screening modalities. Furthermore, the use of tissue-engineered (TE) models and finite element simulations may offer a promising route for generating well-controlled in vitro and synthetic EIS data. These approaches could help overcome current challenges such as data imbalance and limited lesion diversity and support the development of more physically informed machine learning frameworks. However, we acknowledge that translating findings from in vitro and simulated environments to in vivo clinical settings remains a challenge. Nonetheless, these strategies may complement in vivo studies and represent an important direction for future work.

## Conclusions

In conclusion, this study demonstrates the potential feasibility of employing CNN-based models with EIS for the diagnosis of oral cancer. The results show that CNNs show promising ability to differentiate between normal, high-risk, and cancerous oral tissues. Moreover, the proposed threshold adjustment method significantly enhances diagnostic sensitivity, achieving a clinically requisite balance between sensitivity and specificity. These findings imply that, with further data augmentation and clinical validation, CNN-based EIS holds promise as a potential non-invasive tool for early detection of oral cancer, thereby contributing to future advancements in oral lesion screening, pending further validation. The limitation of this study lies in the insufficient amount of human data, therefore, in future research, more data will be collected and used for model training to validate the robustness of the proposed method.

## Electronic supplementary material

Below is the link to the electronic supplementary material.

Supplementary Material 1

## Data Availability

Data Availability Data cannot be shared publicly for confidentiality reasons. However, the interested reader can contact the corresponding author to request access to the data. Requests for data may be granted to eligible parties on reasonable requests and with the completion of any required prerequisites, such as a data use agreement.
