# Supplementary material for: Deep learning-based electrical impedance spectroscopy analysis for malignant and potentially malignant oral disorder detection
Source: Sci Rep. 2025 Jun 3;15:19458. doi: 10.1038/s41598-025-05116-8 (PMC12134277; doi:10.1038/s41598-025-05116-8)
Supplement: Supplementary file 1 — Supplementary Material 1 [file 41598_2025_5116_MOESM1_ESM.docx]

**Supplementary data**

**Supplementary Table S1** Convolutional layer setting.

| 1 | Input 1x28 |
| --- | --- |
| 2 | 10 1x3 convs, stride [1,1], BN + ReLU |
| 3 | 10 1x3 convs, stride [1,1] |
| 4 | Output 1x28 |

The deep learning models trained in this study were implemented using the PyTorch library based on Python. In Figure 4, the CNN structure consists of two convolutional layers, one input layer, two (or three) hidden layers, and one output layer [1].

During training and testing of DL models, each EIS data will be input to the convolutional layers in the form of a matrix with dimensions 1 row by 28 columns as $\left[ x_{r1},x_{r2},\ldots,x_{r14},x_{im1},\ldots,x_{im14} \right]$ where $x_{rn}$ and $x_{imn}$ denotes the real and imagery part of $nth$ signal of each EIS case. In the convolution operation, multiple 1 row by 3 columns sized convolution kernels will be convolved with the input along the column direction. In PyTorch, after each convolutional layer, there will be a ReLU layer and a BatchNorm (BN) layer added to increase the nonlinearity of the model [2]. The setting of a convolutional layer is shown in Table S1. After convolutional operation, the output size is recovered to 1 row by 28 columns and then input to the next fully connected layer (input layer).

The input layer and the hidden layer are composed of fully connected (FC) layers, and the specific number of neurons and layers are specified explained in the experiment part. Each FC is followed by a ReLU and a BN layer for nonlinear operations.

**Supplementary Table S2** Testing AUC results of Task 1.

| Test_AUC | Mean ± SD [%] | |
| --- | --- | --- |
|  | 1:1 0.1 | 0.1:1 0.1 |
| Model_2l_2c_1 | 0.971 ±0.024 | N/A |
| Model_2l_2c_2 | 0.971 ±0.023 | **0.977 ±0.015** |
| Model_3l_2c_2 | **0.979 ±0.015** | N/A |

**Supplementary Table S3** Task 1 testing ACC results.

| Test_ACC | Mean ±SD [%] | |
| --- | --- | --- |
|  | 1:1 0.1 | 0.1:1 0.1 |
| Model_2l_2c_2 | N/A | 0.912 ±0.039 |
| Model_3l_2c_2 | **0.922 ±0.034** | N/A |

**Supplementary Table S4** Task 1 specificity and sensitivity analysis on testing data.

| Model | Specificity | Sensitivity |
| --- | --- | --- |
| Model_2l_2c_2 | 0.949 ±0.018 | 0.812 ±0.036 |
| Model_3l_2c_2 | **0.952 ±0.021** | **0.840 ±0.033** |

**Supplementary Table S5** Testing AUC results of Task 3.

|  | Mean ±SD [%] | | |
| --- | --- | --- | --- |
|  | 1:1 0.1 | 0.1:1 0.1 | 0.1:1 0.01 |
| Model_2l_2c_1 | 0.890 ±0.113 | N/A | N/A |
| Model_2l_2c_2 | **0.907 ±0.097** | **0.903 ±0.092** | 0.884 ±0.114 |

**Supplementary Table S6** Testing ACC results of Task 3.

| Model_2l_2c_2 | |
| --- | --- |
| 0.1:1 0.1 | 1:1 0.1 |
| 0.904 ±0.041 | 0.905 ±0.045 |

**Supplementary Table S7** Specificity and Sensitivity analysis of Task 3.

| Model_2l_2c_2 | Specificity | Sensitivity |
| --- | --- | --- |
| 0.1:1.0 0.1 | 0.978 | 0.592 |
| 1.0:1.0 0.1 | 0.968 | 0.643 |

**Reference**

[1] A. Krizhevsky, I. Sutskever, and G. E. Hinton, ‘ImageNet classification with deep convolutional neural networks’, *Commun. ACM*, vol. 60, no. 6, pp. 84–90, May 2017, doi: 10.1145/3065386.

[2] H. Liu, A. Brock, K. Simonyan, and Q. V. Le, ‘Evolving Normalization-Activation Layers’, Jul. 17, 2020, *arXiv*: arXiv:2004.02967. Accessed: Mar. 05, 2024. [Online]. Available: http://arxiv.org/abs/2004.02967
